# Short Chain Fatty Acids (SCFAs)-Mediated Gut Epithelial and Immune Regulation and Its Relevance for Inflammatory Bowel Diseases

**DOI:** 10.3389/fimmu.2019.00277

**Published:** 2019-03-11

**Authors:** Daniela Parada Venegas, Marjorie K. De la Fuente, Glauben Landskron, María Julieta González, Rodrigo Quera, Gerard Dijkstra, Hermie J. M. Harmsen, Klaas Nico Faber, Marcela A. Hermoso

**Affiliations:** ^1^Laboratory of Innate Immunity, Program of Immunology, Institute of Biomedical Sciences, Faculty of Medicine, Universidad de Chile, Santiago, Chile; ^2^Department of Gastroenterology and Hepatology, University of Groningen, University Medical Center Groningen, Groningen, Netherlands; ^3^Program of Cell and Molecular Biology, Faculty of Medicine, Institute of Biomedical Sciences, Universidad de Chile, Santiago, Chile; ^4^Inflammatory Bowel Diseases Program, Department of Gastroenterology, Clínica Las Condes, Santiago, Chile; ^5^Department of Medical Microbiology, University of Groningen, University Medical Center Groningen, Groningen, Netherlands; ^6^Department of Laboratory Medicine, University Medical Center Groningen, University of Groningen, Groningen, Netherlands

**Keywords:** SCFAs, IBD, immune cells, IECs, intestinal mucosa, dysbiosis

## Abstract

Ulcerative colitis (UC) and Crohn's disease (CD), collectively known as Inflammatory Bowel Diseases (IBD), are caused by a complex interplay between genetic, immunologic, microbial and environmental factors. Dysbiosis of the gut microbiome is increasingly considered to be causatively related to IBD and is strongly affected by components of a Western life style. Bacteria that ferment fibers and produce short chain fatty acids (SCFAs) are typically reduced in mucosa and feces of patients with IBD, as compared to healthy individuals. SCFAs, such as acetate, propionate and butyrate, are important metabolites in maintaining intestinal homeostasis. Several studies have indeed shown that fecal SCFAs levels are reduced in active IBD. SCFAs are an important fuel for intestinal epithelial cells and are known to strengthen the gut barrier function. Recent findings, however, show that SCFAs, and in particular butyrate, also have important immunomodulatory functions. Absorption of SCFAs is facilitated by substrate transporters like MCT1 and SMCT1 to promote cellular metabolism. Moreover, SCFAs may signal through cell surface G-protein coupled receptors (GPCRs), like GPR41, GPR43, and GPR109A, to activate signaling cascades that control immune functions. Transgenic mouse models support the key role of these GPCRs in controlling intestinal inflammation. Here, we present an overview of microbial SCFAs production and their effects on the intestinal mucosa with specific emphasis on their relevance for IBD. Moreover, we discuss the therapeutic potential of SCFAs for IBD, either applied directly or by stimulating SCFAs-producing bacteria through pre- or probiotic approaches.

## Introduction

Inflammatory Bowel Diseases (IBD), comprising mainly ulcerative colitis (UC) and Crohn's disease (CD), are characterized by chronic and recurrent inflammation in the gastrointestinal tract. Symptoms such as diarrhea, abdominal cramps, weight loss, fatigue, anemia, and extra-intestinal signs (arthralgia or arthritis among others), have major impact on quality of life. Both disorders are characterized by intermittent active (mild, moderate, or severe) and inactive periods (remission or quiescence). The incidence and prevalence of UC and CD have increased worldwide in the last 50 years, especially in developing/Western countries. IBD is a result of a complex interplay between genetic, immunologic, microbial, and environmental factors, making development of a subtype-specific treatment a challenging task. Thus, increasing efforts are ongoing to develop personalized therapies to induce remission of these diseases and improve the patient's quality of life (1–3).

The gut microbiome has gained increasing attention as a factor that controls intestinal homeostasis in healthy individuals. Various lifestyle and environmental factors, such as hygiene and the use of antibiotics, together with the consumption of a “Western diet” low in fiber and high in fat and sugar are associated with an imbalanced intestinal microbiota, or dysbiosis, which may lead to chronic inflammation and metabolic dysfunction (4, 5). The perturbation of the microbiota can create an inflammatory environment in the gastrointestinal tract, altering intestinal homeostasis (6, 7), as seen in IBD. Innate and adaptive inflammatory cells infiltrating the lamina propria(LP) can produce pro-inflammatory cytokines (such as IFN-γ, IL-17, TNF-α, or IL-1β) exacerbating the inflammatory process, causing epithelial damage and intestinal and extra-intestinal symptoms (3, 8). However, it remains unclear whether dysbiosis is a cause or a consequence of IBD (9).

The intestinal microbiome of a healthy individual is a balanced community of different microorganisms, including bacteria, bacteriophages, viruses, archaea, and fungi (10). The bacterial community participates in maintaining intestinal homeostasis through the “training” of the immune system and inhibiting growth of pathogens and pathobionts (11, 12). Intestinal inflammatory responses are modulated by the gut microbiome. This may go either way, e.g., IL-10 deficient mice show less severe chronic bowel inflammation in germ-free (GF) conditions (13, 14), while acute chemically-induced colitis is exacerbated in GF mice compared to mice with a normal microbiome (15). Also in humans the importance of microbiota in controlling inflammation, for instance when a bowel segment is excluded from the fecal stream leading to diversion colitis/pouchitis (16). Particularly important appear to be bacterial species that feed on non-digestible dietary fibers (DF) and produce metabolites that exert positive effects on the intestinal mucosa; examples being short-chain fatty acids (SCFAs), mainly acetate, propionate, and butyrate. Butyrate is a primary energy source for colonocytes and also maintains intestinal homeostasis through anti-inflammatory actions (17, 18). At the cellular level, SCFAs can have direct or indirect effects on processes such as cell proliferation, differentiation, and gene expression. They may be absorbed by passive diffusion, but uptake by intestinal epithelial cells is greatly enhanced by dedicated transporters, e.g., the monocarboxylate transporter 1 (MCT1; encoded by *SLC16A1*) and the sodium-coupled monocarboxylate transporter 1 (SMCT1; encoded by *SLC5A8*). Moreover, SCFAs act as ligands for G-protein coupled receptors (GPCRs), including GPR109A, GPR43, and GPR41, thereby activating anti-inflammatory signaling cascades (5, 19–24). Importantly, IBD patients not only show reduced levels of dominant SCFAs-producing bacteria (like *Faecalibacterium prausnitzii* and *Roseburia intestinalis*) in intestinal mucosa and feces, but the actual steady state levels of SCFAs herein also appear to be lower compared to healthy controls (25–29).

IBD patients show dysbiosis and loss of microbiome diversity, most prominently in CD patients (28), and the associated alterations in SCFA levels might be restored by new treatment strategies. One method currently evaluated is fecal microbiota transplantation (FMT) obtained from healthy donors, which effectively induces remission in UC (30). However, long-term durability and safety still needs to be established. Other strategies for microbiome restitution are the use of prebiotics or fiber-rich diets combined with probiotics, as SCFAs-producing single microorganism or combinations may alleviate symptoms by improving butyrate levels.

Here, we aim to provide an overview of microbial SCFAs production in the intestine and their effect on intestinal cells and the immune response. Moreover, gut microbiome changes in IBD are reviewed and how they are related to impaired intestinal SCFAs production and associate to cell metabolism and signaling pathways controlling mucosal homeostasis. Finally, the therapeutic potential of SCFAs for IBD will be discussed; either applied directly or through activation of SCFAs-producing bacteria by prebiotic or probiotic approaches.

## Short Chain Fatty Acids (SCFAs) Bacterial Production

### Intestinal SCFAs Production

SCFAs are carboxylic acids with aliphatic tails of 1–6 carbons of which acetate (C2), propionate (C3), and butyrate (C4) are the most abundant produced by anaerobic fermentation of dietary fibers (DF) in the intestine. DF were defined in 2009 as “*carbohydrate polymers with three or more monomeric units, which are neither digested nor absorbed in the small intestine of humans*” by the Codex Alimentarius (“Food Code”) Commission (CAC), which is part of the Food and Agriculture Organization of the United Nations and the World Health Organization (FAO/WHO) Food Standards Programme (31). From the non-digestible DF, the main substrates for bacterial fermentation and SCFA production are resistant starch (RS), inulin, oat bran, wheat bran, cellulose, Guar gum, and pectin. In particular, RS is an important source for butyrate production (32). Bacteroidetes (gram-negative) and Firmicutes (gram-positive) are the most abundant phyla in the intestine, with members of the Bacteroidetes mainly producing acetate and propionate, while Firmicutes mostly produce butyrate in the human gut (33, 34).

**Figure 1:**
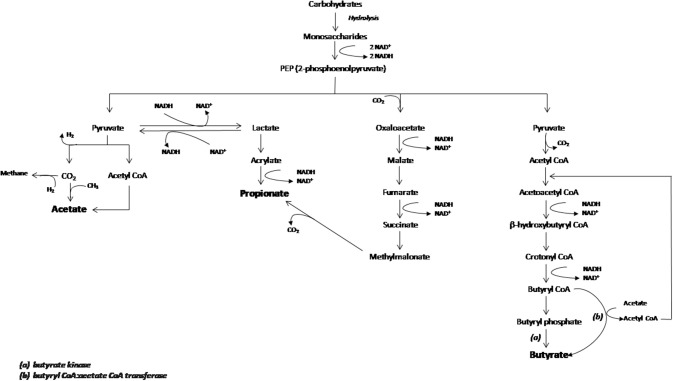
Schematic representation of carboydrates fermentation pathways that lead acetate, propionate and butyrate production. The main enzymes involved in the butyrate production are indicated as (a) butyrate kinase and (b) butyryl CoA:acetate CoA transferase. Figure adapted from den Besten et al. (35).

Butyrate and propionate formation in the gut occurs mainly from carbohydrate metabolism in glycolysis, but can also take place from organic acids and amino acids metabolism (34). In addition, acetate is the most abundant SCFA in the gut produced from acetyl-CoA derived from glycolysis and can also be transformed into butyrate by the enzyme butyryl-CoA:acetyl-CoA transferase (Figure 1) (35–38).

Quantification of human intestinal SCFAs only provides steady state levels and may not accurately reflect the level of bacterial production as most SCFAs produced in the colonic lumen (90–95%) are absorbed by the gut mucosa (39). Nevertheless, the analysis of SCFAs in fecal samples is used as an approximation of gut levels, since excreted SCFA concentrations are associated with RS enriched diets (substrates of SCFAs-producing bacteria), inferring the relationship between intestinal SCFAs production and fecal levels (40, 41).

SCFAs concentrations (expressed as molality or molarity) have been measured in intestinal tissue and fecal samples from individuals of different ethnicity (42–45). In the human gastrointestinal tract, the highest SCFA concentration is found in colon at a molar ratio of approximately 60:20:20 for acetate:propionate:butyrate (Table 1), taken from *post mortem* human subjects (42).

**Table 1:** SCFAs concentration in human samples.

	**Samples**	**Concentration**	**Cohort**	**References**
Total SCFA	Cecum (mmol/kg)	131 ± 9	English	(42)
	Descending colon (mmol/kg)	80 ± 11		
	Ileum (mmol/kg)	13 ± 6		
	Portal blood (mM)	0.375 ± 0.070		
	Liver (mM)	0.148 ± 0.42		
	Peripheral blood (mM)	0.079 ± 0.022

**Table:** 

	**Samples**	**Acetate**	**Propionate**	**Butyrate**	**Cohort**	**References**
SCFAs variants	Colon molar ratio	60	20	20	English	(42)
	Fecal concentration (μmol/g = mmol/kg)	209.7 ± 14.0	93.3 ± 5.3	176.0 ± 16.0	Malaysian	(44)
	Fecal concentration (mM)	87 (58.4–114.9)	21.6 (16.5–27.2)	14.7 (10.3–24.6)	Belgian	(45)
	Fecal concentration (mM)	39.9–56.1	12.8–23.6	12.2–19.0	Japanese, Chinese and Australian	(43)

In contrast, the molar ratio of acetate:propionate:butyrate in fecal samples of healthy subjects varies among cohorts, while propionate and butyrate content are similar with an estimated concentration of 20 and 15 mM, respectively (Table 1).

SCFAs concentrations were found higher in proximal colon (around 70–140 mM) than distal colon (around 20–70 mM) in pigs, although this varies depending on the intake of DF (43).

Finally, SCFAs levels in other tissues such as liver or blood (Table 1) are much lower than in the intestine (42), demonstrating that SCFAs signaling, uptake and/or metabolism mainly occur at the intestinal mucosa. However, detection of extra intestinal levels implies that these metabolites have systemic functions, as established for central nervous system autoimmunity (46).

### Main SCFAs Producers

The main butyrate producing-bacteria in the human gut belong to the phylum Firmicutes, in particular *Faecalibacterium prausnitzii* and *Clostridium leptum* of the family *Ruminococcaceae*, and *Eubacterium rectale* and *Roseburia* spp. of the family *Lachnospiraceae* (33, 34). In addition, sugar-and/or lactate-utilizing bacteria produce butyrate from lactate and acetate, such as *Eubacterium hallii* and *Anaerostipes* spp. (33).

Still, the list of butyrate-producing bacteria may be much longer as members of Actinobacteria, Bacteroidetes, Fusobacteria, Proteobacteria, Spirochaetes, and Thermotogae are potential butyrate producers according to the genes they express, including those that encode enzymes that synthesize butyrate, such as butyryl-CoA dehydrogenase, butyryl-CoA transferase and/or butyrate kinase (47). Moreover, apart from butyrate, the production of other SCFAs is mediated by bacteria such as *Bifidobacterium* species (belonging to the Phylum Actinobacteria) that produce acetate and lactate during carbohydrate fermentation (48). Also, the mucin-degrading bacteria *Akkermansia muciniphila* (Phylum *Verrucomicrobia*) produces both propionate and acetate (34, 49).

The main butyrate-producing bacteria are anaerobes, including the Bacteroidetes and Clostridia, and the low O_2_ concentrations in the colon create a favorable niche for them. Moreover, butyrate absorbed and metabolized by the epithelium consumes (local) O_2_ and thereby stabilizes the hypoxia-inducible factor (HIF, a transcription factor coordinating barrier protection) (50). These data are consistent with studies demonstrating that streptomycin-treated mices how relapse of gastroenteritis by *Salmonella* (51) as well as the expansion of potentially pathogenic *E. coli* (52). The susceptibility due to the depletion of anaerobic bacteria (induced by antibiotics) is associated to a reduction in butyrate levels, thus promoting an aerobic environment and the expansion of aerobic bacteria such as *Salmonella typhimurium* (51, 52). In addition, depletion of butyrate-producing bacteria by antibiotic treatment reduces the intracellular butyrate/PPARγ signaling, increasing iNOS and nitrate levels, favoring Enterobacteriaceae expansion (52).

## SCFAs Functions in the Intestinal Mucosa

**Figure 2:**
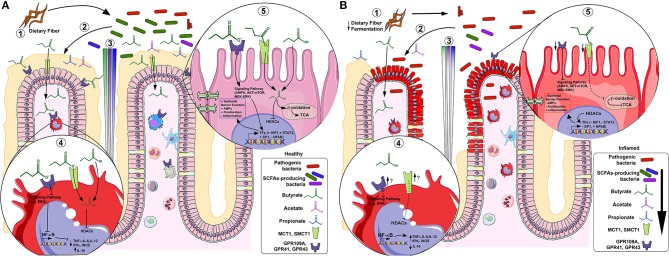
SCFAs in healthy **(A)** and inflamed **(B)** colonic mucosa. In healthy mucosa, (1) bacterial fermentation of dietary fiber (DF) by SCFAs-producing bacteria (e.g., *F. prausnitzii*), increases luminal content of butyrate (green), propionate (blue) and acetate (purple) (2), forming a gradient along the crypt. In lamina propria (LP) macrophages under acute inflammatory stimulus (4), butyrate inhibits histone deacetylases (HDACs) thus; NF-κB-induced pro-inflammatory mediators (e.g. TNF-α, IL-6, IL-12 and iNOS) expression whereas increases anti-inflammatory mediators (e.g., IL-10). In colonocytes (5), butyrate is β-oxidized to Acetyl-CoA and constitutes the main source of energy by entering the TCA cycle. Alternatively, butyrate initiates signaling pathway activation (or repression) by GPCRs and/or directly inhibits HDACs, thus activating (e.g., HIF-1, STAT3 and SP1) or repressing (e.g., NF-κB) transcription factors (TFs), increasing epithelial barrier function, antimicrobial peptides (AMPs) production, cell proliferation and decreasing inflammation. In inflamed mucosa as IBD, (1) a decreased fermentation of DF by low levels of SCFAs-producing bacteria (e.g., *F. prausnitzii*) (2), reduces SCFAs luminal content (3). In LP inflammatory macrophages (4), butyrate-GPCRs activation and -HDACs inhibition are downregulated, thus, there is uncontrolled NF-κB-induced pro-inflammatory mediators' expression (e.g. TNF-α, IL-6, IL-12 and iNOS) and decrease of anti-inflammatory mediators (e.g., IL-10), although it appears that the inflammation increasesthe GPCRs and transporters expression. In inflamed colonocytes (5), butyrate uptake and oxidation are decreased and GPCRs and transporters are also downregulated. This contributes to decreased epithelial barrier integrity, AMPs production, cell proliferation and increased inflammation.

In the intestinal mucosa; acetate, propionate and butyrate exert beneficial effects over intestinal epithelial cells (IECs) and immune cells through induction of intracellular or extracellular processes (see Figure 2 for more details). SCFA may permeate through the cell membrane by passive diffusion (19). However, their absorption is greatly enhanced by two different solute transporters, the proton-coupled monocarboxylate-transporter 1 (MCT1/*SLC16A1*) and the sodium-coupled monocarboxylate-transporter 1 (SMCT1/*SLC5A8*) (20, 21). Alternatively, SCFA may activate signaling pathways via at least 3 different GPCRs: GPR41 (free fatty acid receptor 3; *FFAR3*), GPR43 (free fatty acid receptor 2; *FFAR2*), and GPR109A (hydroxycarboxylic acid receptor 2; *HCAR2*). These receptors are pertussis toxin (PTX)-sensitive, thus coupled to G_i−o_ type G proteins mediate the inhibition of adenylyl cyclase whilst activating AMP-dependent and, to a lesser extent, the phospholipase C (PLC) pathway. In addition, GPR43 mediates G_q_ protein whilst signaling through the PLC pathway (5, 22–24) (see Table 2 for transporters and GPCRs tissue and cell expression).The main cellular functions of SCFAs in the intestinal mucosa are described below.

**Table 2:** SCFAs transporters and receptors.

	**Ligands**	**Tissue or cell expression**	**Species**	**References**
**TRANSPORTERS**				
MCT1	**Butyrate**, lactate, pyruvate	Distal colon> proximal colon>ileum>jejunum	H	(53)
		Transverse colon>ascending and descending colon>sigmoid colon	H	(54)
		Cecum>colon>stomach and small intestine	M, R	(55, 56)
		Monocytes, granulocytes and, lymphocytes	H	(57, 58)
		Peritoneal macrophages	M	(59)
SMCT1	**Butyrate** **>** **propionate** > lactate >> **acetate**	From terminal ileum to distal colon	M	(55)
		Distal colon>proximal colon and ileum	H, M	(60)
**G-PROTEIN COUPLED RECEPTORS**				
GPR41	**Propionate** = pentanoate = **butyrate**>**acetate**>formate	Adipose tissue > PBMCs, pancreas, spleen, and placenta	H	(22)
		Monocytes, neutrophils and, monocyte-derived DCs	H	(61, 62)
GPR43	**Acetate** = **propionate** = **butyrate**>pentanoate> hexanoate>formate	Intestinal epithelium	M, H	(6)
		Monocytes, neutrophils and PBMCs and B/T-lymphocytes	H	(22, 61, 63)
		Treg (colon > spleen and MLN) and colonic myeloid cells	H	(64)
GPR109A	D-beta-hydroxybutyrate, **butyrate** and nicotinic acid	Adipose tissue (>lung, adrenal gland, and spleen)	H, M	(65)
		Colon (> ileum, jejunum, and duodenum)	H, M	(66)
		Monocytes, monocyte-derived DCs, DCs (blood, splenic and colonic), macrophages (splenic and colonic), and BMDM	H, M	(62, 67, 68)

*PBMCs, peripheral blood mononuclear cells; DCs, dendritic cells; BMDM, bone marrow-derived macrophages; H, human; M, mouse; R, rat*.

*Main SCFAs (acetate, propionate, and butyrate) are highlighted in bold font*.

### SCFAs and Cell Proliferation

Small intestinal IECs show a reduced proliferative activity and turnover in GF or antibiotic-treated specific pathogen-free (SPF) mice (69). This is reversed, however, when GF or SPF mice treated with Gram-positive commensal bacteria or a mix of SCFA (acetate, propionate and butyrate) (69). These observations demonstrate the role of the commensal microbiota and their products maintaining the intestinal homeostasis and IECs turnover. In line, SCFAs regulate epithelial gene expression involved in energy metabolism (e.g., lipid metabolism), and promote the development of mouse intestinal organoids (69, 70), further reinforcing their role in supporting epithelial cell proliferation.

On the other hand, recently it was shown that butyrate appears to have a different effect on intestinal stem/progenitor cells, inhibiting their proliferation and delaying wound repair through the transcription factor Foxo3 (71). This suggests that SCFAs and particularly butyrate has cell type-specific effects in the intestinal epithelium and may be linked to local SCFA concentrations, where differentiated IECs in the villus are exposed to higher concentrations of microbial metabolites compared to the stem cells in the crypt (71). As part of the maintenance of intestinal homeostasis, and in contrast to the effect on IECs in healthy conditions, SCFAs suppress cancer cell proliferation and induce apoptotic cell death (72). Moreover, SCFAs induce autophagy in colon cancer cell lines (HCT-116, SW480, and HT-29) (73, 74), as a protective response against apoptosis. These observations are interesting in the context of host-microbe interaction in healthy and colorectal cancer (75) though this is beyond the scope of this review.

### SCFAs and the Epithelial Barrier

The SCFA butyrate promotes the epithelial barrier function, being a main stabilizing mechanism for HIF-1 (as previously mentioned in Main SCFAs producers). Both HIF-1α expression and butyrate levels are reduced in antibiotic-treated or GF mice, but HIF-1α expression is restored after butyrate supplementation (76). Importantly, butyrate induces the barrier function (measured by FITC-dextran flux) in T84 cells, but not in the absence of HIF-1β, demonstrating a crucial role for HIF-1 in maintaining barrier integrity (76). Furthermore, butyrate promotes the epithelial barrier function through induction of genes encoding tight-junctions (TJ) components and protein reassembly through the activation of other transcription factors, including STAT3 and SP1 (Table 3). As a result, butyrate maintains and/or increases transepithelial electrical resistance (TEER) in human colonic Caco-2 and T84 cells (77–80), rat small intestine cdx2-IEC cells (81) and small intestine porcine IPEC-J2 cells even when exposed to inflammatory conditions (82). Such effect can also be achieved in Caco-2 cells by supplementing a supernatant of CD microbiota with probiotic *Butyricicoccus pullicaecorum* 25-3^T^ or a mix of six butyrate-producers when compared to the treatment of CD microbiota-supernatant alone (87). These results reinforce the evidence that the metabolite butyrate restores intestinal barrier function in inflammatory conditions *in vitro* (82), being relevant in the context of IBD, where intestinal epithelial healing is an important therapeutic target. Another important mechanism involved in the epithelial barrier function is the production of antimicrobial peptides (AMPs) by IECs. Recently it was shown that the expression of the AMPs RegIIIγ and β-defensins is strongly impaired in Gpr43 KO mice, while butyrate/Gpr43 activation induced AMP production in *in vitro, ex vivo*, and *in vivo* models (88). This indicates that the effects of SCFAs are not only restricted to inter-epithelial junctions, but also involve regulation of epithelium/luminal bacteria interaction through the production of AMPs as first line defense effectors against pathogens.

**Table 3:** Impact of SCFAs on intestinal homeostasis.

**Cell type**		**Model**	**SCFAs**	**Effect**	**Mechanism**	**References**
Epithelial cells	Cell lines	Caco-2	Butyrate 2 mM	**↑** TEER, ZO-1, occludin	Activation of AMPK Inhibition of MLCK/MLC2 pathway and phosphorylationof PKCβ2	(77, 78)
			Butyrate 5 mM	↑ TEER, claudin-7, claudin-2	Not determined	(79)
		Caco-2, T84	Butyrate 5 mM Propionate 20 mM	↑ TEER, ↓ Claudin-2	Induction of IL-10RA through STAT3 activation and HDAC inhibition	(80)
		Cdx2-IEC	Butyrate 4 mM	↑ TEER, claudin-1, ZO-1, occludin	Induction of Claudin-1 transcription through SP1	(81)
		IPEC-J2	Butyrate 1 mM	↓ LPS impairment of intestinal barrier ↑ Claudin 3 and claudin 4	Activation of Akt/mTOR signaling	(82)
		CCD841, KM12L4, and HCT116	Butyrate 1 mM	Blockade of LPS-induced NF-κB	Activation of GPR109A	(66)
	Primary cells	Colon culture	Butyrate 0.5 mM	↑ IL-18 mRNA and protein	Activation of GPR109A	(68)
		Mouse small intestine organoids	Acetate, propionate and butyrate 5 mM	↑*Fiaf*, Hdac3, Hdac5 ↓ Gpr43, Pparγ	Not determined	(70)
			Acetate, propionate and butyrate 0.5 mM	↑Promotion of organoids development	Activation of GPR41 or GPR43 and MEK-ERK signaling	(69)
Immune cells	Cell lines	RAW 264.7	Sodium butyrate (NaB), sodium phenylbutyrate (NaPB) and sodium phenylacetate (NaPA) 0.5–1 mM	↑ IL-10 ↓ IFN-γ-induced iNOS, TNF-α, IL-6	Inhibition of NF-κB and ERK signaling pathways	(83)
	Primary cells	Human LP macrophages	Butyrate enemas 100 mM	↓ Inhibition of NF-κB translocation	Not determined	(84)
		Human monocytes	Acetate, propionate and butyrate 0.2–20 mM	↑ PGE_2_ ↓ MCP-1, IL-10	Activation of PTX-sensitive GPCRs	(61)
		PBMC	Acetate, propionate and butyrate 0.2–20 mM	↓ LPS-induced TNF-α and IFN-γ	Not determined	
		Human monocyte-derived DCs	Propionate and butyrate 1 mM	↓ LPS-induced chemokines and cytokines (CXCL9-CXCL11), cytokines (IL-6 and IL-12p40)	Not determined	(62)
		Mouse LP macrophages and BMDM	Butyrate 0.1–2 mM	↓ LPS-induced mediators NO, IL-6, IL-12p40	Inhibition of HDACs	(67)
		Mouse DCs	Butyrate 0.125–2 mM	↑ Foxp3*^+^*CD4^+^ T cells	Inhibition of HDACs	(85)
		Mouse LP macrophages and DCs	Butyrate 0.5 mM	↑ Foxp3*^+^*CD4^+^ T cells	Activation of GPR109A	(68)
		Mouse T cells	Propionate butyrate 0.1 mM	↑ Foxp3 and IL-10 in naïve CD4^+^ T cells	Activation of GPR43 and Inhibition of HDACs	(64, 86)

### SCFAs as Energy Source

Butyrate is the main energy source of colonocytes (48), as demonstrated for primary colonocytes from the human ascending and descending colon, which consume more than 70% of oxygen due to butyrate oxidation (89). Interestingly, an energy-deprived state (reflected by decrease of enzymes involved in tricarboxylic acid cycle) leads to lower ATP levels and, ultimately autophagy, observed in GF mice colonocytes. Recolonization of GF mice with butyrate-producing bacteria and butyrate treatment of GF colonocytes *ex-vivo*, increases oxidative phosphorylation and suppresses autophagy to normal levels (17), implying the importance of host-microbe interaction in energy metabolism of colonic epithelium.

### Anti-inflammatory Effects of SCFAs

Apart from the physiological functions of SCFAs detailed above, they also exert anti-inflammatory effects in intestinal mucosa by histone deacetylases (HDACs) inhibition and activating the GPCRs present in IECs and immune cells (Table 3). In IECs models, butyrate suppresses lipopolysaccharide (LPS)-induced NF-κB activation via GPR109A *in vitro* in colonic cell lines and *ex vivo* in mouse colon (66). In addition, the acetate/GPR43 pathway stimulates potassium efflux and hyperpolarization in HT-29 and NMC460 colonic cells leading to NLRP3 inflammasome activation (90). In concordance with these observations, IL-18 is activated in colonic epithelial cells from mice fed on high fiber diet following dextran sulfate sodium (DSS)-colitis (90). These results confirm an important role of GPR109A and GPR43 activation by SCFAs in controlling inflammation and promoting epithelial repair in the colon. Interestingly, butyrate enhances the MCT1 surface expression in the colonic cell line C2BBe1 in a GPR109A-dependent manner (91), suggesting a “cooperative role” between these proteins in mediating butyrate effects.

With respect to innate immune functions, SCFAs induce prostaglandin E_2_ release and expression of the anti-inflammatory cytokine IL-10 through PTX-sensitive GPCRs, thereby inhibiting inflammatory responses in human monocytes (61). The molecular mechanism involved in pro-inflammatory mediator suppression (e.g., LPS-induced chemokines and cytokines) by SCFAs has not been completely determined in other human/mouse mononuclear cell models (62, 67, 83). In addition to the anti-inflammatory effects of the microbial metabolism of dietary fibers to SCFAs, it is important to note that such fibers may also modulate the intestinal immune system directly. For instance, DF pectin (with low degree of methyl esterification) blocks the pro-inflammatory Toll-like receptor (TLR) 2-1 pathway in human dendritic cells (DCs) and mouse macrophage cell lines as well as in an ileitis *in vivo* mouse model (92). These results show that DF regulates inflammatory reactions in intestinal immune and epithelial cells not only after being metabolized by gut bacteria.

The inhibition or reversal of the immune cell inflammatory profile (M1-like macrophages toward a M0-like non-polarized) or polarization toward M2-like anti-inflammatory macrophages is a therapeutic target in the context of IBD. In this way, butyrate effects on mouse IL-4-polarized M2 macrophages are contradictory, as it enhances or suppresses *Arg-1* and *Ym1* expression (M2-profile markers) (93, 94). Therefore, clarification is needed of SCFAs effect on macrophage polarization including the evaluation of human *ex vivo* models and other markers that could ensure stronger conclusions.

Also, SCFAs (mainly butyrate) have inhibitory effect over HDACs activity promoting histone acetylation, affecting gene regulation of cell proliferation, differentiation, and inflammatory response, contributing to intestinal homeostasis and cancer protection (67, 95–99). HDACs regulate innate immunity pathways, controlling myeloid cell differentiation and inflammatory response mediated by TLR- and IFN-inducible gene expression (100). Furthermore, the use of HDACs inhibitors (e.g., valproic acid) reduce disease severity and inhibit colonic proinflammatory cytokines (TNF-α, IFN-γ, and IL-6) in experimental murine colitis (101). These results are promising in regard to the search of alternatives for IBD therapy and support the importance of butyrate as an HDAC inhibitor.

## Regulation of SCFA Transporters and Receptors in the Intestinal Mucosa

### Physiological Regulation of Transporters and GPCRs by Ligands

In line with the SCFAs production in the gut, prominent expression of MCT1 and SMCT1 is observed in the colon of humans, mice and rats, while much lower levels are detected in ileum (see Table 2). Effective absorption of SCFAs from the gut lumen requires an apical location of MCT1, however, depending on experimental approaches it has also been detected in basolateral membranes of the human colonic epithelium (53–55). SMCT1, on the other hand, has been mainly detected in the apical membranes in proximal and distal colon (55, 60), as well as in the ileal enterocytes (55, 60, 102). Interestingly, GF mice show a decreased expression of SMCT1 in colon and ileum, which is recovered by recolonization of the gut with bacteria (102).

MCT1 is considered to be the primary transporter for butyrate uptake in intestinal epithelial cells and its expression is induced by butyrate and fermentable carbohydrates, as demonstrated in *in vitro, ex vivo, and in vivo* models, as described below.

Butyrate induces *SLC16A1* (encoding MCT1) mRNA expression coinciding with enhanced protein expression in Caco-2 cells and in the apical membrane of human colonic AA/C1 and C2BBe1 cells (91, 103, 104). In addition, the direct effect of butyrate studied in *ex vivo* pig colonic mucosa culture showed an up-regulation of *SLC16A1* mRNA expression (103).

These *in vitro*/*ex vivo* observation are confirmed *in vivo* where gastrointestinal levels of MCT1 are enhanced in rats receiving a pectin-supplemented diet, particularly in the apical membrane of colonic mucosa, and increases the transepithelial flux of butyrate (56, 91). Similar observations were made in pigs, where *SLC16A1* mRNA levels increased in cecal and colonic mucosa after dietary supplementation with RS compared to digestible starch (DS) (41). In contrast, *SLC5A8* mRNA levels (encoding SMCT1) were not affected by RS or DS diet (41), showing a specific regulation of MCT1 expression by RS in comparison to SMCT1. On the other hand, a high-protein diet (with or without fermentable carbohydrates) lead to a reduction in colonic MCT1/*SLC16A1* expression in pigs without affecting the butyrate levels (103). This was accompanied by an induction of *TNF-*α, *IL-8*, and *IFN-*γ mRNA expression, suggesting that the inflammatory environment influences in the expression of the butyrate transporter. Similar as described for SCFA transporters, epithelial GPR43 and GPR109A expression in mouse and human intestinal mucosa is related to the proximity to bacterial metabolite production in the colonic lumen (6, 66, 68), suggesting that the levels of these proteins are controlled by their own substrates. In line, GPR109A protein and gene levels are reduced in the ileum and colon of GF mice compared to conventional mice, recovering their normal levels after bacterial re-colonization (102). Moreover, GPR43 expression is reduced in intestinal mucosa of mice fed a “Western-like diet” high in fat and sugar (6). These observations suggest that a reduction in colonic SCFAs as a result of deficiency in specific bacteria or a high fiber diet leads to down-regulation of these SCFA-sensitive GPCRs. In contrast, intestinal mucosal levels of *FFAR2* and *FFAR3* (encoding GPR43 and GPR41, respectively) were not different in pigs fed either a DS- or a RS-containing diet (41), suggesting that dietary fiber does not regulate the gene expression of its metabolite-sensing receptors in these animals. Future studies need to address whether this is truly a species difference or may be caused by experimental differences.

Taken together, it appears that particularly SCFA transporters in the intestinal mucosa, especially MCT1, are highly regulated by their substrates in healthy/non-inflammatory conditions, while this is less well-established for the SCFA-sensing GPCRs. The effect of inflammatory conditions on these mediators of SCFA uptake and signaling is described next.

### Interaction of SCFA Uptake and Signaling With the Intestinal Mucosa in the IBD Context

Among the deregulations detected in the intestinal mucosa of IBD patients, it has been found that the *SLC16A1* gene and MCT1 protein expression is reduced in inflamed mucosa of UC and CD patients (105–107). This may be direct effect of the inflammation or caused by a reduction in butyrate-producing bacteria (see for more details Microbiome changes in CD and UC in relation to SCFAs-producers). In addition, butyrate uptake, and oxidation is inhibited in UC patients compared to healthy individuals (106). Most notably, a significant inverse correlation is observed between butyrate uptake/oxidation and the Mayo endoscopic subscore and Geboes histological score (106). In particular, genes encoding enzymes involved in butyrate metabolism/oxidation (such as *ACSM3, ACADS, ECHS1, HSD17B10, ACAT1, ACAT2, ABAT, ALDH1A1, ALDH2, ALDH9A1, EHHADH, HADHA, HMGCL*, and *PDHA1*) are down-regulated in inflamed mucosa of UC patients (105–108), revealing a specific inflammation-driven gene regulation in the intestine. Interestingly, gene expression of *ACSM3, ACADS, ECHS1, HSD17B10* and *ACAT2* (all enzymes involved in butyrate oxidation), but not *SLC16A1*, increased in mucosa of UC patients that responded to infliximab (human anti-TNF-α antibody) therapy (although only *ACSM3* mRNA levels were higher after therapy than in healthy controls) (106). This suggests that butyrate oxidation is impaired by mucosal inflammation and butyrate supplementation alone would be insufficient to regain homeostasis (106). Hence, these results show that inflammation is tightly linked to the inhibition of genes related to SCFAs uptake and metabolism.

The pro-inflammatory cytokine TNF-α inhibits butyrate oxidation in normal colonic mucosa culture (109), reinforcing the role of inflammatory mediators as part of the intestinal SCFA uptake regulation. Similar observations were made *in vitro* in intestinal HT-29 and (IEC)-6 cell lines showing that inflammatory cytokines inhibit butyrate uptake (60), oxidation and MCT1/*SLC16A1* expression (105). Additionally, MCT1 was downregulated in Caco-2 cells and *ex vivo* porcine colonic tissue culture, exposed to TNF-α (103).

Regulation of MCT1 expression has mostly been studied in IECs, although it also modulates immune cell functions (Table 2). Interestingly, pro-inflammatory stimuli like lipopolysaccharide (LPS) and TNF-α induce *Slc16a1* mRNA and protein expression in mouse peritoneal and J774.1 macrophages, suggesting inflammatory macrophages are sensitive to butyrate (59), but possible respond differently than intestinal epithelial cells. However, more studies are needed to understand how MCT1 is regulated in inflammatory macrophages and its implications for IBD, as they are innate immune cells exacerbating inflammation in intestinal mucosa.

In CD, GPR43 protein expression was lower in ileum of patients either in acute/active or in the quiescent/remissive phase when compared to control subjects (6), suggesting that CD-specific factors are involved in the downregulation of this SCFA receptor, where inflammation seems not to be a crucial determinant.

Animal models have demonstrated the importance of the SCFA/GPCR pathway in IBD. Acute and chronic DSS-induced colitis leads to higher disease activity and colonic inflammation in *Gpr43* KO mice compared to WT littermates, as characterized by increased histological score, neutrophil infiltration together with TNF-α and IL-17 protein levels in the colonic mucosa (15, 90, 110).

Interestingly, high fiber diet or acetate/GPR43 activation suppresses colonic inflammation through NLRP3 inflammasome or cytokine/mediator regulation in DSS-treated GF and WT mice, but not in *Gpr43* KO mice (15, 90, 110), indicating that GPR43 mediates the anti-inflammatory effects of SCFAs in intestinal mucosa. In the same way, LPS-induced TNF-α secretion in mouse-derived peripheral blood mononuclear cells (PBMCs) was suppressed by acetate and reversed by an anti-GPR43 antibody, confirming that acetate/GPR43 signaling mediates anti-inflammatory effects (110). In support of a role for GPR43 in the prevention of intestinal inflammation, mice treated with a GPR43 agonist appear less susceptible to DSS-induced colitis than WT controls (6).

However, not all GPR43-focussed studies appear to give consistent results. A study by Sina et al. reported that *Gpr43* KO mice actually showed less colonic mucosal damage and inflammatory cell infiltration after acute or chronic DSS exposure compared to WT littermates (111). Future studies need to address whether these apparent contradicting results may be explained by the use of different DSS concentrations, time of treatment and/or transgenic mouse services.

Propionate and butyrate treatment increases the chemotactic migration of *ex vivo*-cultured polymorphonuclear leukocytes (PMN) from WT, but not from *Gpr43* KO mice, suggesting that GPR43 activation is relevant in PMN recruitment (111). These observations support the fact that GPR43 activation by SCFAs is important in mounting prompt immune responses.

*Gpr41* and *Gpr43* KO mice show an impaired immune response when exposed to ethanol-induced gut barrier disruption, 2, 4, 6-trinitrobenzene sulfonic-acid (TNBS)-induced colitis, or oral infection with the mucosal pathogen *Citrobacter rodentium*, which is characterized by a decreased neutrophil frequency and expression of inflammatory-associated genes (112). The activation of the acetate/GPCRs pathway accelerates the immune response to *C. rodentium* infection in WT mice demonstrating that IECs mediate the fast immune response dependent on GPR41 and GPR43 activation (112). These observations reveal differences between colonic inflammation models in GPCR KO mice, as acute DSS-induced colitis is characterized by a T_H1_-T_H17_ immune response and in chronic phase is predominantly T_H2_-mediated (113). In TNBS-induced colitis the immune response can be T_H1_, T_H17_, or T_H2_ depending on the mouse strain (113, 114), with *C. rodentium* infection inducing a T_H1_ immune response (115).

As described for *Gpr43* deficient mice, *Gpr109a* KO mice are more susceptible to chemically-induced colonic inflammation and inflammation-associated colon cancer (68). However, the butyrate/GPR109A pathway activates colonic homeostasis by suppressing inflammation in colonocytes (mediated by IL-18 expression and NLRP3 inflammasome activation) (68, 90) and LP macrophages / DCs by differentiating *naïve* T cells to Foxp3^+^ Treg cells and IL-10-producing T cells in WT, but not in *Gpr109a* KO mice (68).

Among the multiple factors involved in IBD pathogenesis, the imbalance between Treg and T effector cells has been the subject of considerable attention to improve IBD therapy. Therefore, in addition to Treg induction mediated by butyrate-induced macrophages and DCs (68), propionate also directly stimulates Treg proliferation and function through GPR43 and HDAC inhibition (64). Also, propionate and butyrate induce colonic Treg differentiation from *naïve* CD4^+^ T cells upregulating *Foxp3* transcription through histone acetylation (85, 86).

In addition, butyrate increases IL-10 production by *ex vivo*-differentiated human Tregs with GPR43-agonists further increasing the suppressive capacity of human Tregs (116), reinforcing the previous evidence of tolerance induction by SCFA in animal models.

As a side note, it is important to mention that depending on the SCFA concentration and cytokine milieu the effects can be 2-fold, either stimulating IL-10-producing T and Treg expansion or T *naïve* differentiation into effector T cells (expressing T-bet transcription factor and IFN-γ; *T*_*H*1_*cells*, or IL-17; *T*_*H*17_) independent of GPR41, GPR43, or SMCT1, but dependent on direct HDAC inhibitor activity (117, 118).

These findings generate new research questions in IBD patient's therapy, such as what is the best formulation of a DF-enriched diet to induce gut immune tolerance? or what is the effect of a high fiber diet or SCFA supplementation on Treg function in IBD patients within an acute or chronic phase? In summary, pharmacokinetic studies, high fiber diet design, and another approach need to be explored to clarify novel therapeutic options for IBD.

Mononuclear cells and neutrophils are innate immune cells mediating the protection against pathogens through recognition and elimination of antigens that cross the epithelial barrier and cytokine/chemokine secretion, thus activating the adaptive immune response. In these cells, expression of SCFA-activated GPCRs is induced by inflammation; thus sensitivity to potential anti-inflammatory actions of SCFAs is increased. Examples are LPS from *E. coli* O55:B5 increases *Gpr109a* mRNA levels in mouse macrophages (119), and also *GPR43* mRNA expression induced by TNF-α, GM-CSF (63) and TLRs (Toll-like receptors) ligands (61, 63) in human monocytes. Moreover, the effect of *E. coli* LPS on *GPR43* mRNA expression in human monocytes may be strain-dependent, as *E. coli* O55:B5 LPS induces *GPR43* mRNA expression (63) whereas *E. coli* O127:B8 LPS does not (61).

Taken together, these studies provide strong evidence for the role of SCFAs/GPCRs (particularly GPR43 and GPR109A), in maintaining colon integrity by inducing mucosa healing and suppressing inflammation. These are relevant therapeutic targets for numerous diseases, but in particular for IBD.

## Microbiome Changes in CD and UC in Relation to SCFAs-producers

Various changes occur in the intestinal mucosa of IBD patients in active or quiescent status compared to healthy individuals, one being the composition and function of the microbiota, a change often referred to as dysbiosis. In general, dysbiosis in IBD patients is associated with a decrease in the number of SCFAs/butyrate-producing bacteria, in particular members of the phylum Firmicutes. In addition, more specific studies show that a decrease in *F. prausnitzii*, a butyrate producing-bacteria from the *Clostridium* cluster IV, is a hallmark of active IBD patients, as reviewed previously covering different populations (25, 26, 120–122). Additionally, CD appears to have a more pronounced dysbiosis than UC, with lower diversity, altered composition and an unstable microbial community (28). Thus, CD and UC are being recognized as distinct diseases even at the microbiome level.

At the species level, alterations in other butyrate-producing species have been detected in UC patients, such as *Roseburia intestinalis* and *Roseburia hominis* (25, 45). Alternatively, stool samples of CD patients show an increase of *Ruminococcus gnavus* and decrease of *F. prausnitzii, Bifidobacterium adolescentis, Dialister invisus*, an uncharacterized species of Clostridium cluster XIVa, and other SCFAs-producing bacteria (*Blautia faecis, Roseburia inulinivorans, Clostridium lavalense*, and *Bacteroides uniformis*) (27, 29).

The microbiome diversity is affected by geography, ethnicity and lifestyle even in the healthy population (123), which also includes the abundance of SCFAs/butyrate-producing bacteria. However, the “environmental” factors in IBD remain unclear. Reported dysbiosis in IBD patients from different populations might be due to sample size, patient selection criteria or genetic heterogeneity, therefore, further studies are required to clarify differences in microbiome diversity among IBD patients.

As a consequence of the reduction in SCFAs-producers, SCFA levels are often found to be decreased in fecal samples of IBD patients. One study showed that acetate and propionate, but not butyrate, are reduced in fecal samples of UC patients (45). In another study, a reduction of butyrate and propionate in stool samples of IBD patients was found (44). Similarly, a low content of n-butyrate, iso-butyrate and acetate was detected in feces from patients with severe UC. The reduction in SCFAs levels might be related to disease activity, as a higher n-butyrate level was detected in UC patients in remission compared to ones with active disease (25).

## Therapeutic Approaches of SCFAs in IBD and Diversion Colitis

SCFAs are considered a promising supplementary treatment in the current clinical management of active IBD patients and diversion colitis. Different approaches, including enemas of butyrate and/or mixtures of SCFAs (acetate, propionate, and butyrate) have resulted in diverse clinical outcomes (16, 124, 125).

The direct effects of butyrate or mixtures of SCFAs in enemas showed clinical and histological improvement in active UC patients and diversion colitis (84, 125–127). At the molecular level, butyrate enemas decrease NF-κB nuclear translocation in LP macrophages in tissue sections from distal UC patients (84), as well as in LPS-induced cytokine expression and NF-κB activation in LP mononuclear cells and PBMCs from CD patients (128).

Alternatively, SCFAs enemas (100 ml of 80 mM acetate, 30 mM propionate, and 40 mM butyrate twice a day for 6 weeks) produced clinical remission only in a subset of UC patients (129). Butyrate enemas (60 ml of 100 mM once daily for 20 days) do not affect daily symptoms score, stool consistency and frequency (Bristol scale), and oxidative stress in UC patients in clinical remission, although they have a small effect on inflammation parameters (130). Moreover, no endoscopic or histological changes were observed in diversion colitis patients treated with SCFAs enemas (60 ml of 60 mM acetate, 30mM propionate, and 40 mM butyrate twice a day for 2 weeks) (131).

Inconsistent effects of SCFAs intervention in murine models undergoing colonic inflammation have been reported. For example, SCFAs enemas did not prevent or reduce intestinal damage in TNBS-induced colitis in rats (132), while butyrate reduced colonic mucosal damage and serum inflammatory cytokines (IL-6, TNF-α, and IL-1β) in DSS-treated mice (93). In contrast, butyrate did not revert/prevent DSS-induced intestinal damage in mice exposed to antibiotics (67). Similarly, butyrate was less effective in eliciting an anti-inflammatory response in the TNBS-induced colitis mouse model, vs. an injection of live *F. prausnitzii* or *F. prausnitzii* supernatant, while they both induced IL-10 and decreased IL-12 and TNF-α (133).

Interestingly, oral treatment with the spent medium of a culture of the SCFA-producer *Clostridium butyricum* (“supernatant”) decreased DSS-induced colonic mucosal damage (134). These contradictory effects of butyrate or SCFAs might be species-specific or due to the colitis model (DSS vs. TNBS), commensal bacteria depletion, butyrate dosing and route of administration. Still, these results suggest that, by itself, butyrate or SCFAs are probably not as effective as administrating direct live SCFAs-producing bacteria to the mucosa. As mentioned earlier, there needs to be a constant production and delivery of SCFA to the mucosa to have anti-inflammatory effects. Nevertheless, anti-inflammatory effects of SCFAs seem (also) to be directed to immune cells. Therefore, the success of SCFAs in restoring intestinal mucosa homeostasis might be achieved by enriching or recovering SCFAs-producing bacteria through the use of pre- or probiotics.

### Use of Prebiotics for SCFAs Production in IBD

The definition of prebiotic is “*a substrate that is selectively utilized by host microorganisms conferring a health benefit*” (135). Typically, these substrates are not digested in the human small bowel, thus promoting selective growth of beneficial bacteria in the colon (136). It is therefore sensible to explore the possible therapeutic role of different supplementary DF as substrates for gut bacteria and SCFA production in order to suppress inflammatory pathways in IBD patients, animal, and *in vitro* models.

A 4- and 12-weeks “intervention” with an oat bran-supplemented diet resulted in an increase of fecal butyrate concentrations and a decrease of abdominal pain or reflux in UC patients (137). Moreover, a double-blind pilot trial demonstrated that oral inulin (oligofructose)-supplementation was well-tolerated by UC patients, with active disease and decreased dyspeptic symptoms and, more importantly, a reduction in fecal calprotectin, as an important marker of intestinal inflammation (138). In contrast, the use of prebiotics has been associated to side effects in CD patients, such as abdominal pain, flatulence, bloating, and diarrhea (139–141). Consequently, the adherence to this supplementation may be compromised in clinical trials, hindering an objective evaluation of the effect of the prebiotic in IBD patients. It remains to be determined whether the difference in patients' response might be related to the specific pathophysiology of both forms of IBD.

Neutrophils may play a dual role in IBD pathophysiology (142). Over activation of neutrophils may cause excessive tissue damage in UC patients, while defective neutrophil recruitment fails to control microorganism invasion in CD, subsequently leading to uncontrolled inflammation and formation of macrophage-containing granulomas in an attempt to contain the microorganism.

This aspect was addressed in CD patients receiving DF supplementation to their enteral nutrition (143), which resulted in an increase in GPR43^+^ neutrophil infiltration when compared to enteral nutrition alone or patients in remission. Thus, prebiotics may be used to improve intestinal neutrophil recruitment.

In pigs fed an RS-supplemented diet, SCFAs concentrations and abundance of butyrate- (*F. prausnitzii*) or propionate-producing (*Propionibacterium, Veillonella, Phascolarctobacterium*) bacteria were increased in the luminal part of cecum and colon, while potentially pathogenic bacteria (*Escherichia coli* and *Pseudomonas* spp.) were decreased (41). Similarly, a high fiber diet protects mice against DSS-induced colitis, increasing protective Bacteroidetes (families *Porphyromonadaceae* and *Rikenellaceae*) and Firmicutes (family *Lachnospiraceae*), compared to a zero fiber diet (90).

Recently, also other prebiotics have been tested to promote intestinal SCFA production, including non-digestible dextrin (DEX), α-cyclodextrin (α-CD), and dextran (DXR) that increased acetate and propionate production in an *in vitro* fecal fermentation model of human colonic microbiota (144). Thus, non-digestible fibers may be a complementary therapy for IBD to increase intestinal butyrate production, especially in UC patients, as supporting evidence in animal and *in vitro* models suggests their benefit in promoting SCFAs-producing bacteria. Nevertheless, well-controlled randomized placebo-controlled trials (RCT) are needed to fine tune a prebiotics supplementation plan to manage gastrointestinal tolerance in IBD patients, especially in CD, before rigorously confirming an actual clinical improvement.

### Use of Probiotics for SCFAs Production in IBD

A probiotic is defined as “*live microorganisms that, when administered in adequate amounts, confer a health benefit on the host*” (145). In IBD patients, the potential effect of probiotics in inducing or maintaining remission, showed encouraging benefits mainly in UC, as described below.

Two meta-analyses and systematic reviews of RCT of IBD with probiotics showed that they have significant effects in achieving remission, particularly for VSL#3 (mixture of four strains of *Lactobacillus*, three strains of *Bifidobacterium*, and one strain of *Streptococcus salivarius* subsp. *thermophilus*), being safe and effective in achieving remission in UC patients (146, 147). Moreover, the treatment with the probiotic preparation VSL#3 induced remission, as determined by a decrease in Ulcerative Colitis Disease Activity Index (UCDAI) in 50–53% UC patients with mild to moderately active disease (148, 149). In addition, VSL#3 combined with *Lactobacillus* have a significant effect in achieving clinical response in children with IBD (146). In an alternative approach, UC patients benefitted from a *Lactobacillus* probiotic when combined with prebiotics (146). Similarly, an oral treatment with the non-pathogenic *Escherichia coli* strain Nissle 1917 (EcN) (for 12 months), reduced relapses of UC patients in clinical remission, as compared to the standard treatment with mesalazine (150). Also, *Bifidobacterium infantis* 35,624 supplementation (for 6 weeks) reduced plasma C-reactive protein levels and tended to decrease IL-6 levels in mild to moderately active UC under treatment with mesalazine, compared to placebo-supplemented patients (151).

So far, probiotic treatments have not shown a significant effect in inducing or maintaining remission of active or quiescent CD, or in preventing relapse of CD after surgically-induced remission (146, 147). However, probiotics evaluated in these studies were not butyrate-producing bacteria. Interestingly, a recent proof-of-concept study explored the effect of six butyrate producers (*B. pullicaecorum* 25-3^T^, *F. prausnitzii, Roseburia hominis, Roseburia inulinivorans, Anaerostipescaccae*, and *Eubacterium hallii*) in an *in vitro* fed batch system that simulates the mucus- and lumen-associated microbiota. A co-culture of these bacteria with fecal microbiota derived from CD patients with active disease showed increased butyrate production and improved epithelial barrier function *in vitro* (87).

These results encourage the exploration of pre- and probiotic therapies for specific SCFAs/butyrate production in restoring intestinal homeostasis and providing resolution and remission in IBD patients. Such approaches may complement alternative strategies to modulate microbiota, such as fecal microbiome transplantation (FMT), which has generated inconsistent results so far. As such, a detailed description of FMT is outside the scope of this review. As promising these results seem, more robust pre-clinical and further RCT studies are still necessary to test safety and efficacy of new SCFAs- or butyrate-producing bacteria (mixtures) with potential to be tested in association with FMT for reconstituting a healthy microbiome.

## Conclusions and Future Perspectives

IBD is characterized by gastrointestinal dysbiosis, both in patients and in animal models, which particularly impairs SCFA production, thereby restraining energy supply to colonocytes and local control of mucosal inflammation. UC and CD patients show decreased butyrate-producing bacteria, especially *F. prausnitzii*, and consequently, SCFAs are reduced in feces, as well as butyrate uptake and oxidation, a process dependent on the mucosal inflammatory context. Empirical modulation of the microbiota using prebiotics or probiotics can increase SCFAs-producing bacteria *in vitro* and *in vivo*, enriching microbiome diversity in animal models and UC patients, demonstrating clinical and histological improvement. However, limited evidence exists indicating clinical improvement through theses therapeutics in CD patients; nevertheless, supplementation with specific probiotics for butyrate formation may still provide new avenues to manage disease activity. The mechanisms involved in IBD pathophysiology are still not resolved, nor how butyrate regulates inflammation, influences metabolism and transcription in colonic mucosa. Future studies are needed to understand how to specifically modulate the microbiota and thus predict possible responses to therapy with personalized strategies in intestinal inflammation.

## Author Contributions

DPV wrote most of the review. MD and GL contributed to writing and correcting the manuscript. MG, RQ, GD, HH, KF, and MH participated reviewing and critically correcting the manuscript. KF and MH contributed to manuscript structure and supervised the work.

### Conflict of Interest Statement

GD has received unrestricted grants from Abbvie and Takeda, is on advisory boards for Mundipharma and Pharmacosmos, and has received speaker's fees from Takeda and Janssen Pharmaceuticals. The remaining authors declare that the research was conducted in the absence of any commercial or financial relationships that could be construed as a potential conflict of interest.
