# Corrigendum: Short Chain Fatty Acids (SCFAs)-Mediated Gut Epithelial and Immune Regulation and Its Relevance for Inflammatory Bowel Diseases

**DOI:** 10.3389/fimmu.2019.01486

**Published:** 2019-06-28

**Authors:** Daniela Parada Venegas, Marjorie K. De la Fuente, Glauben Landskron, María Julieta González, Rodrigo Quera, Gerard Dijkstra, Hermie J. M. Harmsen, Klaas Nico Faber, Marcela A. Hermoso

**Affiliations:** ^1^Laboratory of Innate Immunity, Program of Immunology, Institute of Biomedical Sciences, Faculty of Medicine, Universidad de Chile, Santiago, Chile; ^2^Department of Gastroenterology and Hepatology, University of Groningen, University Medical Center Groningen, Groningen, Netherlands; ^3^Program of Cell and Molecular Biology, Faculty of Medicine, Institute of Biomedical Sciences, Universidad de Chile, Santiago, Chile; ^4^Inflammatory Bowel Diseases Program, Department of Gastroenterology, Clínica Las Condes, Santiago, Chile; ^5^Department of Medical Microbiology, University of Groningen, University Medical Center Groningen, Groningen, Netherlands; ^6^Department of Laboratory Medicine, University Medical Center Groningen, University of Groningen, Groningen, Netherlands

**Keywords:** SCFAs, IBD, immune cells, IECs, intestinal mucosa, dysbiosis

In the original article, we neglected to include the funder “National Commission for Scientific and Technological Research REDES No. 180134 (PCI)” to MH.

The authors apologize for this error and state that this does not change the scientific conclusions of the article in any way. The original article has been updated.

